# Development of Clinical Risk Scores for Detection of COVID-19 in Suspected Patients During a Local Outbreak in China: A Retrospective Cohort Study

**DOI:** 10.3389/ijph.2022.1604794

**Published:** 2022-09-06

**Authors:** Zhuoyu Sun, Yi’an Guo, Wei He, Shiyue Chen, Changqing Sun, Hong Zhu, Jing Li, Yongjie Chen, Yue Du, Guangshun Wang, Xilin Yang, Hongjun Su

**Affiliations:** ^1^ Department of Epidemiology and Biostatistics, School of Public Health, Tianjin Medical University, Tianjin, China; ^2^ Tianjin Key Laboratory of Environment, Nutrition and Public Health, Tianjin, China; ^3^ Tianjin Center for International Collaborative Research in Environment, Nutrition and Public Health, Tianjin, China; ^4^ Department of Radiotherapy, Baodi Clinical College of Tianjin Medical University, Tianjin, China; ^5^ Department of Ophthalmology, Baodi Clinical College of Tianjin Medical University, Tianjin, China; ^6^ Department of Neurosurgery, Baodi Clinical College of Tianjin Medical University, Tianjin, China; ^7^ Department of Social Medicine and Health Service Management, School of Public Health, Tianjin Medical University, Tianjin, China; ^8^ Department of Tumor, Baodi Clinical College of Tianjin Medical University, Tianjin, China; ^9^ Department of Neurology, Baodi Clinical College of Tianjin Medical University, Tianjin, China

**Keywords:** COVID-19, risk score, local outbreaks, clinical variables, retrospective cohort study

## Abstract

**Objectives:** To develop and internally validate two clinical risk scores to detect coronavirus disease 2019 (COVID-19) during local outbreaks.

**Methods:** Medical records were extracted for a retrospective cohort of 336 suspected patients admitted to Baodi hospital between 27 January to 20 February 2020. Multivariate logistic regression was applied to develop the risk-scoring models, which were internally validated using a 5-fold cross-validation method and Hosmer-Lemeshow (H-L) tests.

**Results:** Fifty-six cases were diagnosed from the cohort. The first model was developed based on seven significant predictors, including age, close contact with confirmed/suspected cases, same location of exposure, temperature, leukocyte counts, radiological findings of pneumonia and bilateral involvement (the mean area under the receiver operating characteristic curve [AUC]:0.88, 95% CI: 0.84–0.93). The second model had the same predictors except leukocyte and radiological findings (AUC: 0.84, 95% CI: 0.78–0.89, Z = 2.56, *p* = 0.01). Both were internally validated using H-L tests and showed good calibration (both *p* > 0.10).

**Conclusion:** Two clinical risk scores to detect COVID-19 in local outbreaks were developed with excellent predictive performances, using commonly measured clinical variables. Further external validations in new outbreaks are warranted.

## Introduction

The outbreak of coronavirus disease 2019 (COVID-19), caused by severe acute respiratory syndrome coronavirus 2 (SARS-CoV-2), emerged in Wuhan, China in December 2019, and has been rapidly spreading worldwide. On 30 January 2020, the World Health Organization (WHO) declared the COVID-19 outbreak a public health emergency of international concern (PHEIC) [1] and, on 11 March 2020, characterized it as a pandemic [2]. Up to 18 July 2022, a total of 562 million cases have been reported across 228 countries and territories, resulting in over 6.37 million deaths [3]. The rapid spread of COVID-19 globally, which has significant morbidity with no proven treatment, presents a huge challenge for healthcare system worldwide.

The number of new cases has been increasing rapidly largely due to the easy transmissibility of the virus, especially the hugely infectious Omicron subvariants BA.4 and BA.5, by milder cases or asymptomatic carriers [4]. The transmission often takes place in clusters, such as families [5], working places or schools [6], and crowded public places (e.g., shopping malls or restaurant) [7–9]. Currently, diagnosis of COVID-19 is mainly dependent on specialized nucleic acid-based quantitative real-time reverse transcription polymerase chain reaction (RT-PCR) testing, which is not readily available in low-resource regions [10]. Even in well-resourced areas with local outbreaks, the mass testing to detect cases in millions of population will be a high demand on nursing and laboratory resources. Given that up to 54% of COVID-19 patients may have an initial negative RT-PCR result [11], repeated testing and longer waiting time for the results (more than 24 hours) in hospitals cause a huge burden on already overcrowded healthcare system. In order to prevent the false-negative results, a combination of RT-PCR with clinical assessment, blood tests and imaging has been recommended to help clinicians in the triage of patients at high risk of COVID-19 [12].

A clinical risk-scoring model is certainly to serve the purpose, which combines demographic and clinical variables to identify COVID-19 cases from high-risk patients who need to undergo the RT-PCR testing. At present, several clinical diagnostic models have been developed, but the quality of these models was inconclusive [13]. In a systematic review of 33 diagnostic models for prediction of COVID-19, Wynants et al. concluded that all those studies were suboptimal due to use of undefined study populations, high risk of model overfitting, inappropriate statistical analyses and poor reporting [13]. In a recent study on development and validation of a clinical risk score, the authors focused on patients confirmed with COVID-19 and the outcome of an intensive care unit admission. Their primary objective was to detect patient at high risk of deterioration, but not to develop an efficient tool to classify patients at high risk of COVID-19 [14]. Thus, the present study aimed to construct a clinical risk score based on socio-demographics, epidemiological contact history, clinical features, laboratory and radiological findings, which can identify COVID-19 patients from high-risk individuals during a local outbreak in China. Given that blood tests and CT scans can be invasive and time-consuming, second risk score was also developed without these variables. The risk score will be important for clinicians to make better clinical decisions and better allocate healthcare resource.

## Methods

### Data Sources

Tianjin is a municipality in Northern China, with 15.6 million inhabitants in 2019. As the fourth largest city in China, Tianjin is made up of 12 central districts, and four suburban districts, including Baodi, Jizhou, Jinghai and Ninghe. As of February 25, 2020, a total of 135 cases were diagnosed in Tianjin, and almost half of them were reported in Baodi (60/135), where a cluster outbreak of COVID-19 related to Baodi Shopping Mall occurred during local transmission of the disease in Tianjin [15]. The detailed tracing of the transmission chain of the cluster outbreak was available elsewhere [7]. Briefly, the first case, an employee of the shopping mall, had a close contact with a patient with undiagnosed infection in other province, and subsequently three other employees were infected but were still on duty after symptom onset. Consequently, six employees and 19 customers were infected, whose exposures were directly linked to Baodi Shopping Mall during 19 to 25 January 2020 (9).

### Cohort Description

The present study reviewed medical records of a retrospective cohort of hospitalized patients with suspected infection admitted to Baodi Hospital, Tianjin (China) during 27 January to 20 February 2020 (the second and third stage of the local outbreak in Tianjin) [16]. Patients were not involved in the recruitment to and conduct of the study. Patients’ medical records were excluded if they were younger than 18 years old, or if their results of SARS-COV-2 nucleic acid testing were unavailable at time of data collection. This study was approved by the Institutional Ethics Review Board of Baodi Hospital, Tianjin (#BDYYLL20200011). Written informed consent was waived due to the use of anonymous data. Reporting of the present study followed the Transparent Reporting of a multivariable prediction model for Individual Prognosis Or Diagnosis (TRIPOD) statement [17] (Supplementary TRIPOD Checklist).

### Diagnosis of COVID-19

In accordance with the WHO interim guidance, the diagnosis of COVID-19 was confirmed by a positive high-throughput sequencing or real-time reverse transcription polymerase chain reaction (RT-PCR) assay for SARS-COV-2 nucleic acid from oropharyngeal specimens [18]. The laboratory work was done in the Center for Disease Control and Prevention (CDC) of Baodi, Tianjin (China).

### Data Collection and Extraction

A trained team of physicians and nurses who were blind to the diagnosis of patients retrospectively reviewed medical records and extracted the required data from the electronic management system of Baodi Hospital, Tianjin. Data were extracted based on clinical knowledge, literature and data availability, which included demographics (age, sex and occupation), exposure history, clinical symptoms, laboratory findings and pulmonary computed tomography (CT) scan findings. Exposure history was collected by investigators from Tianjin Centers for Disease Control and Prevention (CDC), who conducted a face-to-face epidemiological investigation for each patient, and the contact tracking was performed to identify potential infection sources [9]. Based on the rigorous epidemiological investigation, exposure history within 14 days before hospital admission included: 1) had a close contact with confirmed or suspected cases of COVID-19; 2) same location of exposure (e.g., had visited the Baodi Shopping Mall or had a close contact with those who visited the same place); 3) had a history of travel to Wuhan or many places in China (including Wuhan).

Clinical symptoms previously reported to be associated with COVID-19 infection were extracted, involving fever, cough, sore throat, sneezing, runny nose, sputum production and digestive symptoms (diarrhea, nausea and vomiting) [15]. Total counts of leukocyte or lymphocyte were extracted and classified into three groups: 1) being within normal range, 2) increased (leukocyte count >10.0 × 10^9^/L; lymphocyte count >4.0 × 10^9^/L), and 3) decreased (leukocyte count<3.5 × 10^9^/L; lymphocyte: <1.0 × 10^9^/L). The CT imaging features reported in previous studies on COVID-19 were recorded here, including bilateral involvement, lower lobes involvement, ground-glass opacification (GGO), peripheral distribution (the lesions distributed in the outer 1/3 of the lung), multiple small plaques, interstitial changes (incl. interstitial thickening or fibrous stripes), and pulmonary nodules [19]. All CT images were reviewed independently by two radiologists. A third experienced radiologist was consulted if there was a disagreement in interpreting CT findings.

### Statistical Analysis

Characteristics of study participants were summarized by mean ± standard deviation (SD) for continuous variables or by numbers and percentages for categorical variables. Differences of characteristics between patients with or without COVID-19 infection were compared using Student’s t-test, chi-square test or Fisher Exact test where appropriate. Complete-case analysis was used for missing variables (missing values <1% in the present study).

### Development of the Clinical Risk Scores

Binary logistic regression analysis was used to identify potential predictors for COVID-19. Potential predictor variables were selected into the multivariate model if the variables had a *p-value* < 0.20 in the univariate analysis, if they were judged to be of clinical importance, or if they had been consistently reported to be associated with COVID-19 in previous studies. In the multivariate logistic regression, an enter method instead of a stepwise method was used for predictor selection to avoid model over-fitting, with statistically significant variables (*p* < 0.05) being retained in the final prediction model [20]. The prediction model was further translated into clinical risk scores using the equation as below, with $\overset{⌢}{p}$ was defined as the predicted probability of detection of patients with COVID-19.

$$\mathrm{Clinical}\;risk\;score=\mathrm{In}\left(\frac{\overset{⌢}{p}}{1-\overset{⌢}{p}}\right)-\mathrm{intercept}$$

### Internal Validation of the Developed Risk Scores

To avoid over-fitting of the constructed risk score, the shrinkage factor was calculated using (*x* ^2^-k)/*x* ^2^, where *x* ^2^ was the likelihood ratio *x* ^2^ and k was the number of the predictors in the model. A shrinkage factor <0.85 may raise the concern of over-fitting. If this is the case, regression coefficients of the predictors will be adjusted for over-optimism by multiplying the shrinkage factor [21]. The performance of the developed risk score was evaluated by calibration and discrimination measures [17]. Hosmer-Lemeshow chi-square tests and calibration plots were used to test if the observed and expected probabilities of COVID-19 were comparable over the deciles of predicted risk [22]. A *p*-value > 0.10 indicated similarity in the predicted and observed probability, i.e., the internal calibration being acceptable. A 5-fold cross-validation method was performed to evaluate the internal discrimination, using the mean area under the receiver operating characteristic curve (AUC) [23]. Using this approach, the cohort was partitioned into five folds of equal size, with one of them selected as the testing set and the remaining four merged as the training set. This approach was repeated five times to ensure that each fold was selected once as the testing set. An AUC is closer to 100%, indicating that the model’s discrimination is perfect. If discrimination is no better than chance, then the AUC is around 50%. Sensitivity, specificity, positive predictive value (PPV) and negative predictive value (NPV) were further calculated at different cutoff points of the risk scores, for clinical application based on local medical resources. All statistical analyses were carried out using SAS 9.4 (SAS Institute, Inc., Cary, United States), SPSS 24.0 (IBM/SPSS, Inc., Chicago IL), R 3.6.3, and MedCalc 20.112. The differences were considered as statistically significant if *p* < 0.05.

## Results

### Characteristics of Patients in the Study

A total of 336 patients were enrolled in the study, and 56 patients were diagnosed with COVID-19 (Table 1). In total, 56 confirmed cases had received 105 tests for SARS-COV-2 nucleic acid, with an average of 2 tests per patient. The average age of 336 patients were 44.1 ± 15.4 years, ranging from 18–93 years. The majority of patients were females (59.5%) and farmers (56.2%). Of them, 28.9% had a close contact with confirmed or suspected cases, and 32.4% had the same location of exposure (incl. had a history of visit to Baodi Shopping Mall or had a close contact with those who visited the same place during 19 to 25 January 2020). Fever (89.6%), normal levels of leukocyte (82.1%) or lymphocyte counts (78.9%), and CT scan findings suggestive of pneumonia (80.7%) were among the most common clinical features in the patients with suspected infection.

**TABLE 1:** Comparisons between patients with or without COVID-19 in terms of socio-demographic characteristics, exposure history, clinical features and pulmonary CT scan findings in the retrospective cohort study, China, 2020.

Variables	All patients	COVID-19 patients	Non-COVID-19 patients	*p* value
	n (%)	n (%)	n (%)	
Age, years (mean ± SD)	44.1 ± 15.4	49.7 ± 14.7	42.9 ± 15.3	0.003
Sex				
Male	135 (40.5)	22 (39.3)	113 (40.8)	0.834
Female	198 (59.5)	34 (60.7)	164 (59.2)	
Occupation				
Non-farmer	146 (43.8)	31 (55.4)	115 (41.5)	0.057
Farmer	187 (56.2)	25 (44.6)	162 (58.5)	
Exposure history				
Close contact with confirmed or suspected cases	97 (28.9)	40 (71.4)	57 (20.4)	<0.001
Same location of exposure^a^	109 (32.4)	14 (25.0)	95 (33.9)	0.193
Travel to Wuhan or many places in China (including Wuhan)	9 (2.7)	1 (1.8)	8 (2.9)	1.000
Fever (yes)	301 (89.6)	52 (92.9)	249 (88.9)	0.380
Temperature	37.4 ± 0.7	37.5 ± 0.7	37.4 ± 0.7	0.149
±1 Respiratory symptoms (yes)	143 (42.6)	20 (35.7)	123 (43.9)	0.256
Cough	113 (33.6)	18 (32.1)	95 (33.9)	0.796
Sore throat	25 (7.4)	1 (1.8)	24 (8.6)	0.095
Sneezing/runny nose	7 (2.1)	1 (1.8)	6 (2.1)	1.000
Sputum production	87 (25.9)	15 (26.8)	72 (25.7)	0.867
±1 Digestive symptoms (yes)^b^	13 (3.9)	3 (5.4)	10 (3.6)	0.461
Leukocyte count category (10^9^/L)				
Normal (3.5–10.0)	276 (82.1)	46 (82.1)	230 (82.1)	
Increased (>10.0)	42 (12.5)	2 (3.6)	40 (14.3)	0.001
Decreased (<3.5)	18 (5.4)	8 (14.3)	10 (3.6)	
Lymphocyte count category (10^9^/L)				
Normal (1.0–4.0)	265 (78.9)	38 (67.9)	227 (81.1)	
Increased (>4.0)	5 (1.5)	0 (0.0)	5 (1.8)	0.033
Decreased (<1.0)	66 (19.6)	18 (32.1)	48 (17.1)	
Pulmonary CT scan findings (yes)				
Findings of pneumonia	271 (80.7)	54 (96.4)	217 (77.5)	0.001
Bilateral involvement	87 (25.9)	28 (50.0)	59 (21.1)	<0.001
Lower lobes involvement	16 (4.8)	1 (1.8)	15 (5.36)	0.488
Ground-glass opacification	52 (15.5)	18 (32.1)	34 (12.14)	<0.001
Peripheral distribution	6 (1.8)	5 (8.9)	1 (0.36)	<0.001
Multiple small plaques	20 (6.0)	4 (7.1)	16 (5.71)	0.756
interstitial changes	18 (5.4)	2 (3.6)	16 (5.71)	0.748
Pulmonary nodules	7 (2.1)	0 (0.0)	7 (2.50)	0.606

a This category included individuals who had visited the Baodi Shopping Mall or had a close contact with those who visited the same place during January 19 to 25, 2020.

b Digestive symptoms included diarrhea, nausea and vomiting.

Abbreviations: COVID-19, coronavirus disease 2019; CT, computed tomography.

COVID-19 patients were older than non-COVID-19 patients. The proportion of subjects with a history of a close contact with confirmed or suspected cases was higher in patients with COVID-19 than those without it. More patients in the COVID-19 group had reduced leukocyte or lymphocyte count. CT scan findings of pneumonia, bilateral involvement, GGO, peripheral distribution were more common among the confirmed cases than their counterparts. No significant differences were observed between the two groups in terms of sex, occupation, fever, respiratory symptoms and digestive symptoms.

### Predictor Selection and Construction of the Clinical Risk Scores

Based on results from the univariate analysis and clinical relevance, 19 variables were included in the multivariate logistic regression, involving age, sex, occupation, close contact with confirmed or suspected cases, same location of exposure, fever, temperature, ≥1 respiratory symptoms, cough, sore throat, sneezing/runny nose, sputum production, ≥1 digestive symptoms, leukocyte count category, lymphocyte count category, pulmonary CT findings of pneumonia, bilateral involvement, ground-glass opacification and peripheral distribution. After predictor selection, seven variables remained to be significant predictors of COVID-19, including age, close contact with confirmed or suspected cases, same location of exposure, temperature, leukocyte count category, CT findings of pneumonia and bilateral involvement (model 1). The results of 5-fold cross-validation method showed that the same predictors were included in the model 1. Given that blood testing and CT scan imaging can be invasive and time-consuming, the model 2 was constructed using the same predictors except leukocyte counts and CT findings. Temperature was centered around its mean by subtracting the mean (i.e., 37.39) to avoid a large intercept. Parameter estimates and odds ratios of predictors in two models were demonstrated in Table 2. The final risk score of COVID-19 in model 1 and model 2 were constructed as follows:

**TABLE 2:** Parameter estimates and odds ratios of predictors of COVID-19 infection in the retrospective cohort study, China, 2020.

Variable	Model 1				Model 2			
	β	Standard error	OR^a^	95% CI	β	Standard error	OR^a^	95% CI
Age	0.02	0.01	1.02	1.01–1.05	0.03	0.01	1.03	1.01–1.05
Exposure history								
Contact with confirmed cases	3.59	0.59	36.32	11.45–115.26	3.27	0.55	26.37	8.91–78.02
Same location of exposure^b^	1.38	0.60	3.98	1.22–12.98	1.44	0.58	4.24	1.35–13.31
Temperature	0.93	0.31	2.53	1.38–4.63	0.97	0.29	2.65	1.51–4.66
Leukocyte count category (10^9^/L)								
Increased (>10.0)	-0.67	0.91	0.51	0.09–3.04	—	—	—	—
Decreased (<3.5)	1.43	0.67	4.18	1.13–15.53	—	—	—	—
Pulmonary CT scan findings								
Findings of pneumonia	2.61	0.91	13.64	2.28–81.58	—	—	—	—
Bilateral involvement	0.90	0.39	2.45	1.15–5.24	—	—	—	—
Intercept	−7.52	1.23	0.001	—	−4.95	0.76	0.007	—

a All factors were adjusted for each other.

b This category included individuals who had visited the Baodi Shopping Mall or had a close contact with those who visited the same place during January 19 to 25, 2020.

Abbreviations: COVID-19, coronavirus disease 2019; CT, computed tomography; OR, odds ratio; CI, confidence interval.

Model 1: COVID-19 risk score = 0.02×age (years) + 3.59×exposure (close contact, 1 for yes/0 for no) + 1.38×exposure (same location of exposure, 1 for yes/0 for no) + 0.93×(temperature-37.39, Celsius) - 0.67×leukocyte (increased, 0 for normal/1 for decreased/2 for increased) + 1.43×leukocyte (decreased, 0 for normal/1 for decreased/2 for increased) + 2.61×CT (pneumonia) + 0.90×CT (bilateral involvement) + 7.52

Model 2: COVID-19 risk score = 0.03×age (years) + 3.27×exposure (close contact, 1 for yes/0 for no) + 1.44×exposure (same location of exposure, 1 for yes/0 for no) + 0.97×(temperature-37.39, Celsius) + 4.95

**FIGURE 1:**
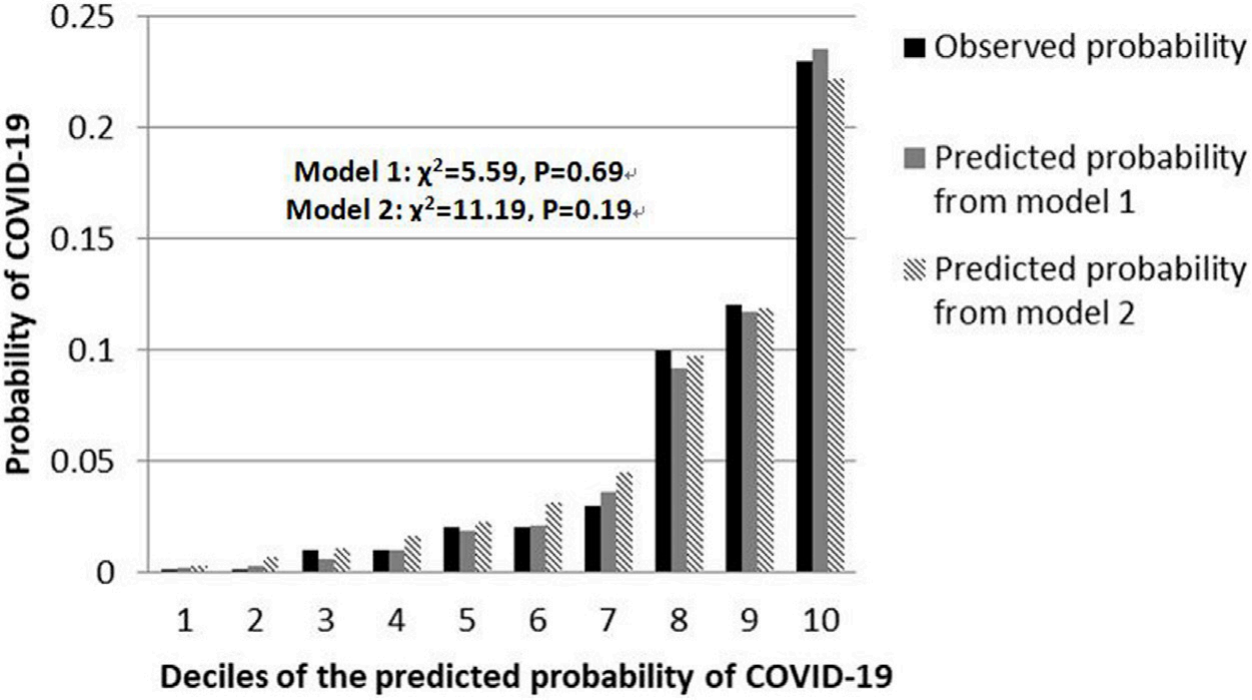
Calibration plots for both risk scores to detect COVID-19 cases in the retrospective cohort study, China, 2020.

**FIGURE 2:**
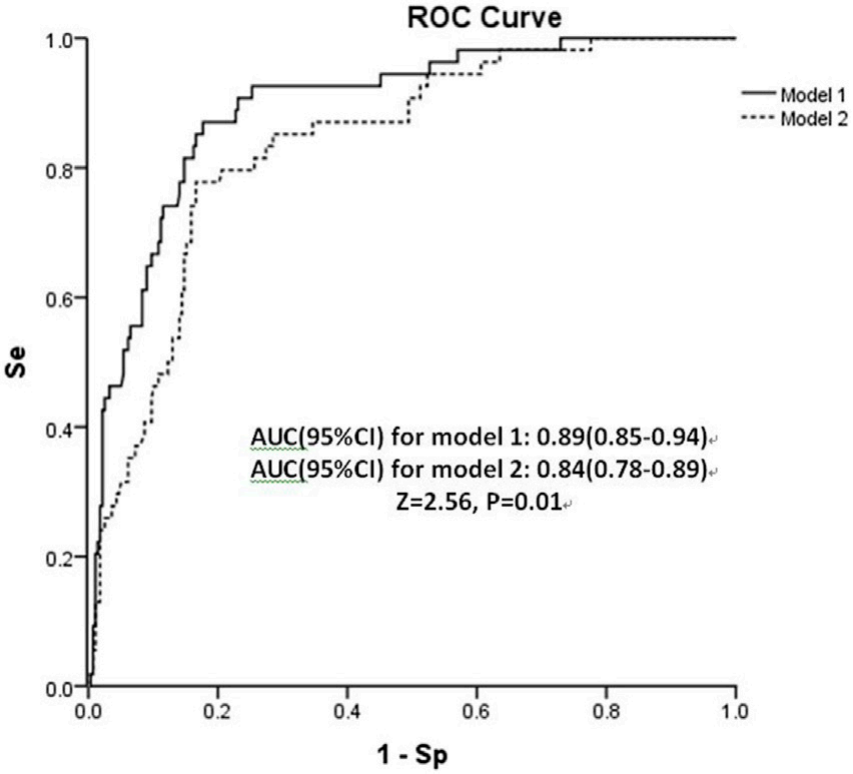
Area under receiver operating characteristic curve (AUC) of both risk scores in the retrospective cohort study, China, 2020. Abbreviation: Se, sensitivity; Sp, specificity.

The shrinkage factor of model 1 and model 2 were 0.93 and 0.94, which was significantly higher than the value of over-fitting criteria (λ = 0.85), indicating that the performance of two models was only overestimated by 7.5% and 5.6%, respectively. The first risk score (model 1) had a good internal calibration, with the predicted probabilities of COVID-19 being similar to the observed probabilities (*x* ^2^ for Hosmer-Lemeshow test = 5.59, *p* = 0.69). The second risk score (model 2) had similar predicted probabilities compared with the observed probabilities (*x* ^2^ for Hosmer-Lemeshow test = 11.19, *p* = 0.19) (Figure 1). Using a 5-fold cross-validation procedure, the mean AUC of the first risk score was 0.88 (95% CI: 0.84–0.93), suggesting an excellent discrimination of the risk score for prediction of COVID-19 in the study. After excluding laboratory and radiological findings, the mean AUC of the second risk score significantly decreased but was quite acceptable (AUC:0.84 95% CI:0.78–0.89, Z = 2.56, *p* = 0.01) (Figure 2).

### Sensitivity, Specificity, PPV and NPV at Different Cutoff Points of the Risk Scores

Because of high transmissibility of COVID-19 and high rates of morbidity and fatality, cutoff points of 3.45 (model 1) and 1.35 (model 2) were recommended to avoid false negative results (Table 3). For instance, at the cutoff point of 3.45 in the model 1 (or 1.35 in the model 2), 100% of COVID-19 patients were identified with a specificity of 27.1% (or 22.4%). With use of these cutoff points, around 76.5%∼80.4% of patients would need to undergo the SARS-CoV-2 nucleic acid testing, without any cases being missed. If 4.40 were set as a threshold in the model 1, 45.8% of patients could avoid the tests, with the missed diagnosis rate of 5.6%. The PPV and NPV were 28.5% and 97.9%, respectively. While a cutoff point of 2.25 in the model 2 might rule out 41.1% of patients to undergo the tests, with a PPV of 26.4% and a NPV of 97.7%.

**TABLE 3:** Sensitivity, specificity, predictive values at different cutoff points of the risk scores in the retrospective cohort study, China, 2020.

Model 1							Model 2						
Risk score cutoff point	Population at or above the value n (%)	Se (%)	Sp (%)	PPV^a^ (%)	NPV^a^ (%)	Predicted probability of COVID-19 (%)	Risk score cutoff point	Population at or above the value n (%)	Se (%)	Sp (%)	PPV^a^ (%)	NPV^a^ (%)	Predicted probability of COVID-19 (%)
1.00	95.8	100.0	2.9	17.1	100.0	0.1	1.00	87.5	100.0	13.4	18.8	100.0	1.9
2.00	92.6	100.0	7.2	17.7	100.0	0.4	1.20	82.4	100.0	19.5	19.9	100.0	2.3
3.00	80.7	100.0	21.7	20.3	100.0	1.1	^b^1.35	80.4	100.0	22.4	20.5	100.0	2.7
^b^3.45	76.5	100.0	27.1	21.5	100.0	1.7	1.40	78.6	98.2	24.2	20.6	98.5	2.8
3.60	74.1	98.2	29.6	21.8	98.8	2.0	1.60	73.2	98.2	31.1	22.2	98.8	3.4
3.80	69.9	98.2	34.3	23.0	98.9	2.4	1.80	69.3	98.2	35.4	23.3	99.0	4.1
4.00	64.9	98.2	40.8	24.9	99.1	2.9	2.00	64.3	94.4	40.4	24.1	97.3	5.0
4.20	58.6	94.4	47.3	26.4	97.7	3.5	2.20	59.8	94.4	45.9	25.9	97.6	6.0
^b^4.40	54.2	94.4	52.7	28.5	97.9	4.2	^b^2.25	58.9	94.4	47.3	26.4	97.7	6.3
4.60	49.7	92.6	57.8	30.5	97.5	5.1	2.35	54.8	90.7	50.5	26.8	96.5	6.8
4.80	45.5	92.6	63.2	33.5	97.7	6.2	2.40	52.7	87.0	53.1	27.1	95.3	7.3
5.00	39.9	92.6	69.7	37.9	97.9	7.5	2.60	47.6	87.0	59.2	29.9	95.8	8.7
5.20	37.5	92.6	72.9	40.6	98.0	9.2	2.80	42.6	85.2	65.3	33.0	95.7	10.6
5.40	35.1	90.7	75.8	42.9	97.6	10.8	3.00	37.8	85.2	71.5	37.4	96.0	12.5
5.60	32.1	87.0	78.3	44.6	96.8	12.8	3.20	34.2	81.5	74.4	38.9	95.3	14.8
5.80	29.5	87.0	81.2	48.1	96.9	15.4	3.40	30.7	79.6	78.3	42.4	95.1	17.8
6.00	27.7	85.2	83.4	50.6	96.6	18.3	3.60	27.7	77.8	82.0	46.3	94.9	20.8

a PPV and NPV were calculated based on the prevalence of COVID-19 in the dataset (i.e. 56/336).

b The suggested cutoff points.

Abbreviation: Se, sensitivity; Sp, specificity; PPV, positive predictive value; NPV, negative predictive value; COVID-19, coronavirus disease 2019.

## Discussion

In this study, two clinical risk scores were developed and internally validated to identify COVID-19 cases from highly suspected patients during a cluster outbreak, based on commonly measured clinical variables. The predictive performances of both risk-scoring models were satisfactory with excellent internal calibration and discrimination. The risk scores constructed can be easily used by clinicians to identify high-risk patients for the SARS-CoV-2 nucleic acid testing, while up to 20%∼40% of them were exempted from the testing.

It is worth noting that some variables can be immediately collected upon hospital arrival, such as socio-demographics, clinical symptoms and contact tracing history; while others such as vital signs, laboratory or radiological tests are time consuming and only available at a later stage. Two parallel prediction models were constructed in our study, one with and the other without laboratory tests and imaging findings. The full model with all seven predictors produced a mean AUC of 0.88 (95% CI: 0.84–0.93), while the simplified 4-predictor model had an AUC of 0.84 (95% CI: 0.78–0.89, Z = 2.56, *p* = 0.01). Consistent with our results, a validation and agreement study evaluated six previously published models and found that models based only on clinical symptoms and/or risk exposures had lower but though acceptable performance values compared with those based on biological parameters and/or radiological findings, highlighting the importance of these parameters to obtain more efficient models [24]. In case of limited testing facilities, the simplified risk score was able to maintain good performance, still being useful as early screening procedure. When testing facilities are sufficient or an immediate level of risk is resulted, it would be more appropriate to perform further examinations to confirm COVID-19 positivity and minimize false negative results.

In the current study, age, contact with confirmed or suspected cases, same location of exposure, temperature, leukocyte count category, CT scan findings suggestive of pneumonia and bilateral involvement were found to be most significant predictors of COVID-19, which were in line with the guideline for COVID-19 diagnosis and literature [13, 19]. The guidelines for diagnosis and treatment of COVID-19 infection, issued by the National Health Commission of the People’s Republic of China, suggested that patients had a high risk of COVID-19 if they had at least two of the following symptoms, in addition to a history of travel to Wuhan or contact with other infected patient: 1) fever and/or respiratory symptoms; 2) imaging features of pneumonia; 3) normal or reduced leukocyte count, or reduced lymphocyte count [25]. Recent published systematic reviews of 21 studies on diagnostic models to detect COVID-19 in patients with suspected infection indicated that the recurrent significant predictors were socio-demographics (age and gender), epidemiological contact history, clinical symptoms (temperature or fever), vital signs, laboratory or biological tests (leukocytes or lymphocytes) and radiological examinations (pneumonia signs on CT scan) [13, 26]. Thirty four prediction models for diagnosis of COVID-19 were proposed based on images, and most of them used CT images [13]. Our findings provided evidence to support that imaging features of pneumonia was the typical appearance of COVID-19 radiologically [13, 25–27].

In the current study, two clinical risk scores were constructed based on a retrospective cohort of patients with suspected infection in a hospital setting during a cluster outbreak. Both risk scores were developed based on commonly measured clinical variables, with excellent internal calibration and discrimination to detect COVID-19 cases from high risk individuals who need to undergo the nucleic acid testing. Our study revealed significant predictors of COVID-19 in a cluster outbreak, which were consistent with the latest systematic reviews [13, 26]. Our findings of two risk scores developed had an implication in terms of applicability in clinical practice. When testing facilities are sufficient, a full model with laboratory and imaging findings can be more effective to detect COVID-19 cases. In case of limited testing resources with a high volume of patients, a simplified risk score can be useful to identify COVID-19 patients at first admission. Moreover, the risk scores constructed can be easily used by clinicians to decide which risk scores would produce the best cut-offs for RT-PCR testing. Hence, in well-resourced areas with good access to healthcare, a risk score of 3.45 in the model 1 (or 1.35 in the model 2) might be set as a threshold, and 76.5% of patients (or 80.4% of patients in the model 2) would receive the nucleic acid tests, with no cases being missed. On the contrary, in areas with local outbreaks or limited diagnostic resources, a cutoff point of 4.40 in the model 1 (or 2.25 in the model 2) might rule out 45.8% of patients (41.1% of patients in the model 2) to undergo the tests, as long as they could be isolated at home to minimize the transmission of COVID-19 to others.

A major limitation of the present study was that the risk score was developed using a small sample of patients in the early phase of COVID-19. Further external validations in independent samples from local outbreaks of COVID-19 are warranted. Nevertheless, our study sample was collected in the early outbreak of COVID-19 and was less likely to be confounded by the use of prevention measures, such as use of various vaccines. Using a 5-fold cross-validation procedure, the same predictors were included in the final model, and the mean AUC of both risk scores was still excellent. Additionally, seven significant predictors of COVID-19 identified in our study were consistent with previous literature [13, 25–27]. Dardenne et al. evaluated and compared diagnostic models for COVID-19 identified in a recently published systematic review, and they found similar results between the first and the second wave in terms of model performances [24]. Theoretically speaking, application of any model in the clinical practice should undergo a thorough investigation and a pilot testing phase. In that case, findings from the current study might be a good initiative to develop and refine them in other settings. Again, Some potential predictors reported in previous studies were unavailable in the current study, such as neutrophil-to-lymphocyte ratio, C reactive protein, and heart ratio [13, 27]. However, considering the practical issue in clinical application, a simple and interpretable model is often preferred. This is a single-center retrospective study, which may limit the generalizability of the risk scores in other places. Multi-center studies with a large sample of patients are urgently needed.

### Conclusion

In the current study, two clinical risk scores to detect COVID-19 in highly suspected patients during a local outbreak was developed based on commonly measured clinical variables, with excellent predictive performances. The risk scores may assist clinicians in screening high-risk patients for COVID-19 with exemption of 20∼40% from further RT-PCR testing, thus better use of limited healthcare resources. However, further external validations using standardized and multi-centered datasets are needed.

## Data Availability

The dataset supporting the conclusion of this article is available upon reasonable request from the corresponding author.
